# HbA_1c_ Measured in the First Trimester of Pregnancy and the Association with Gestational Diabetes

**DOI:** 10.1038/s41598-018-30833-8

**Published:** 2018-08-16

**Authors:** Stefanie N. Hinkle, Michael Y. Tsai, Shristi Rawal, Paul S. Albert, Cuilin Zhang

**Affiliations:** 1 Epidemiology Branch, Division of Intramural Population Health Research, Eunice Kennedy Shriver National Institute of Child Health and Human Development, National Institutes of Health, Bethesda, MD USA; 2 Department of Laboratory Medicine and Pathology, University of Minnesota Medical School, Minneapolis, MN USA; 3 Department of Nutritional Sciences, School of Health Professions, Rutgers University, Newark, NJ USA; 4 Biostatistics Branch, Division of Cancer Epidemiology and Genetics, National Cancer Institute, National Institutes of Health, Bethesda, MD USA

## Abstract

We aimed to examine the prospective association between first trimester HbA_1c_ and gestational diabetes (GDM) and explore the utility of HbA_1c_ for prediction of GDM. We used data from a case-control study within the prospective NICHD Fetal Growth Studies-Singleton Cohort (2009–2013), which enrolled 2,802 women at 12 U.S. clinical centers. HbA_1c_ was measured in GDM cases (n = 107) and matched controls (n = 214) targeted at 8–13, 16–22, 24–29, and 34–37 gestational weeks. We excluded women with HbA_1c_ ≥ 6.5% (48 mmol/mol) at enrollment (n = 3) or who had a hemoglobin variant (n = 6). At 8–13 gestational weeks, women who later developed GDM had significantly higher HbA_1c_ (5.3[standard deviation 0.3]%; 34[4]mmol/mol) than women without GDM (5.1[0.3]%; 32[3] mmol/mol) (P ≤ 0.001); this difference remained significant throughout pregnancy. Each 0.1% (1 mmol/mol) HbA_1c_ increase at 8–13 weeks was associated with an adjusted 22% increased GDM risk (95% confidence interval 1.09–1.36). First trimester HbA_1c_ significantly improved GDM prediction over conventional risk factors (AUC 0.59 vs 0.65; P = 0.04). In conclusion, women who develop GDM may have impaired glucose homeostasis early in or prior to pregnancy, as indicated by their elevated first trimester HbA_1c_. First trimester HbA_1c_ may aid in early identification of at risk women.

## Introduction

Gestational diabetes (GDM) is a common pregnancy complication associated with adverse maternal and fetal outcomes including an increased risk for type 2 diabetes and cardiovascular disease later in life in mothers and an increased risk for macrosomia and obesity in offspring^1^. A recent study reported that GDM associated fetal overgrowth starts early in pregnancy before diagnosis of GDM, potentially demonstrating a need to identify pregnancies with glucose intolerance earlier in pregnancy^2^. HbA1c, a measure of glycated hemoglobin which serves as an indicator of blood glucose control in the prior 3–4 months, may be an avenue for earlier identification of women at risk for GDM. However, while HbA_1c_ is currently used among high-risk women at the first prenatal visit to identify women with overt type 2 diabetes, it is not currently used to screen for GDM.

A few prior studies have examined if HbA_1c_ measured in the first trimester is useful for early predication of GDM^3–6^; however, these studies have been among high-risk populations only^4,5^, evaluated an HbA_1c_ threshold only of 5.7% (39 mmol/mol), corresponding to prediabetes outside of pregnancy^6^, or used GDM diagnosed in the first trimester only as the outcome^3^. Other studies have focused primarily on HbA_1c_ measured in the second trimester or at the time of GDM diagnosis^7–9^. Thus, research remains limited on HbA_1c_ measured in the first trimester and its relation with GDM among a population based sample. This is particularly important as early risk prediction may offer a unique opportunity for earlier interventions. Furthermore, data is lacking presenting normal ranges for HbA_1c_ across pregnancy.

This study aimed to comprehensively examine of HbA_1c_ across pregnancy and its relation with GDM. The first aim was to profile the physiological variation in HbA_1c_ across gestation and examine for differences in women with and without GDM. The second aim of this study was to prospectively evaluate the association between HbA_1c_ levels measured in the first trimester and subsequent risk for GDM diagnosis. The third exploratory aim of this study was to evaluate the predictive utility of using first trimester HbA1c to predict GDM and potentially identify an ideal cut-off for GDM screening in the first trimester.

## Methods

### Study sample

This study was based on a secondary analysis of a GDM case-control study using participants from the *Eunice Kennedy Shriver* National Institute of Child Health and Human Development (NICHD) Fetal Growth Studies-Singleton Cohort, with 2,334 low-risk, pregnancies among non-obese women and 468 pregnancies among obese women (n = 2,802 in total)^10^.Women from four self-identified race-ethnic groups (non-Hispanic white, non-Hispanic black, Hispanic, and Asian/Pacific Islander) were enrolled between gestational weeks 8–13 at 12 U.S. clinical centers (2009–2013). The primary aims of the NICHD Fetal Growth Study were to develop fetal growth standards and thus enrollment was restricted to non-obese women without preexisting chronic diseases or medical conditions, including diabetes before pregnancy or GDM in a prior pregnancy, and without lifestyle risk factors including smoking (n = 2,334). A secondary aim was to examine the etiology of GDM and thus a supplemental cohort of obese women was also recruited (n = 468). The inclusion criteria were less restrictive for the obese cohort and included women who smoked prior to pregnancy, had a hematologic disorder, or had GDM in a prior pregnancy. Complete details on the inclusion criteria are detailed elsewhere^10^. Longitudinal questionnaire data and biospecimens were collected throughout pregnancy and medical record abstraction of routine prenatal exam results and delivery discharge diagnoses reports was completed after delivery. Institutional review board approval was obtained at all participating clinical sites (Christiana Care Health System, Columbia University, Fountain Valley, Long Beach Memorial Medical Center, Medical University of South Carolina, New York Hospital Queens, Northwestern University, St. Peter’s University Hospital, Tufts University, University of Alabama at Birmingham, University of California, Irvine, Women and Infants Hospital of Rhode Island), the data coordinating centers (Clinical Trials & Surveys Corporation and the Emmes Corporation), and the NICHD. All participants provided written, informed consent. All methods were performed in accordance with the relevant guidelines and regulations. The NICHD Fetal Growth Studies follows the data sharing policies of the NICHD and queries regarding data sharing may be sent to the corresponding author.

The current analysis is based on a nested GDM case-control study within the NCIHD Fetal Growth Studies, which included 107 GDM cases and 214 matched non-GDM controls. Two non-GDM controls were selected for each case and matched on maternal age (±2 years), race/ethnicity (non-Hispanic White, non-Hispanic Black, Hispanic, Asian/Pacific Islander), and gestational week of blood collection (±2 weeks). All analyses excluded participants with HbA_1c_ ≥ 6.5% (48 mmol/mol) at enrollment (n = 3), as this is an indicator of overt type 2 diabetes^11^. Furthermore, participants missing HbA_1c_ measurements at all timepoints (n = 1), or who had an abnormal hemoglobin variant such as HbS, HbC, or HbE (n = 6) were excluded. Thus, the final analytic sample included 100 GDM cases and 211 non-GDM controls.

### GDM ascertainment

All women underwent standard clinical care which included a glucose challenge test and/or an oral glucose tolerance test (OGTT), as needed. GDM cases were identified by medical record review of the clinical OGTT results according to Carpenter and Coustan criteria, as currently endorsed by the American Diabetes Association (ADA) and the American College of Obstetrics and Gynecologists (ACOG), of at least two diagnostic plasma glucose measurements at or above the defined thresholds (fasting 5.3 mmol/l, 1-h 10.0 mmol/l, 2-h 8.6 mmol/l, 3-h 7.8 mmol/l)^12,13^. Women without recorded OGTT results, but with ‘medication treated GDM’ recorded on the discharge diagnoses were considered as having GDM (n = 12). A total of 107 women with GDM were identified in the NICHD Fetal Growth Studies.

### Blood Collection

Blood specimens were collected in all participants following a standardized protocol at enrollment at 8–13 gestational weeks and at three additional study visits targeted at weeks 16–22 (fasting), 24–29, and 34–37. The actual date range for the blood collection varied slightly due to some women coming in at a different time for their blood draw than their regular study visit. At enrollment (8–13 gestational weeks), 99.7% of the blood draws were within the targeted range. At visit 1 (16–22 weeks), 91.0% of the blood draws were within the targeted range. At visit 2 (24–29 weeks), 90.3% of the blood draws were within the targeted range. No blood was collected at visit 3. At visit 4 (34–37 weeks), 89.2% of the blood draws were within the targeted range.

### HbA_1c_ measurements

HbA_1c_ was measured in an EDTA whole blood sample that was stored at <−70 °C and thawed immediately before analysis. HbA_1c_ was measured using a non-porous ion Exchange High Performance Liquid Chromatography (HPLC) assay (Tosoh Automated Analyzer HLC-723G8, Tosoh Bioscience, Inc., South San Francisco, CA & Tokyo, Japan). The assay CV was less than 1.16%.

HbA_1c_ was measured in blood collected at enrollment, visit 1, 2 and 4 in all GDM cases and one of the two matched controls. However, for the second of the two non-GDM controls, HbA_1c_ was measured only in samples collected at enrollment and the first visit, before the time GDM is typically diagnosed.

### Covariates

All women underwent a screening ultrasound at enrollment to confirm accurate dating of the pregnancy by last menstrual period, which was then used to calculate gestational weeks at each subsequent visit. At enrollment, women completed detailed questionnaires regarding their medical history and socio-demographic characteristics. Maternal height was measured, and pre-pregnancy weight was self-reported. Pre-pregnancy body mass index (BMI; kg/m^2^) was calculated and categorized as normal weight (18.5–24.9 kg/m^2^), overweight (25.0–29.9 kg/m^2^), or obese (≥30.0 kg/m^2^). Family history of diabetes was classified (yes/no) if a woman’s parents or siblings had diabetes. As part of the inclusion criteria for the main study (primary aim was to define a fetal growth standard), non-obese women who smoked, had GDM in a prior pregnancy or had a hematologic disorder (e.g., chronic anemia, sickle cell disease, low platelets, blood clotting problems) were not eligible for the study. The inclusion criteria for obese women was less restrictive and obese women reported at enrollment their smoking habits in the 6 months prior to pregnancy (yes, no), if they had GDM in a prior pregnancy (yes, no), or if they had a hematologic disorder (yes, no). A three-level variable was created incorporating parity and prior GDM status (nulliparous/parous, no GDM/parous, prior GDM).

### Statistical methods

The bivariate baseline characteristics of GDM cases and controls were compared using binomial/multinomial logistic regression with generalized estimating equations accounting for the matching factors between cases and controls.

#### HbA_1c_ Profile Across Pregnancy

Longitudinal trajectories of the mean HbA_1c_ levels across gestation were plotted by visit according to GDM status. Differences between GDM cases and controls were tested using linear mixed models.

#### Prospective Association between HbA_1c_ and GDM Risk

We examined the prospective association between HbA_1c_ measured in the first trimester and GDM risk as well as the change between HbA_1c_ in the first and second trimesters and GDM risk. We examined for, but did not detect a possible non-linear relation between HbA_1c_ in the first trimester and the odds of GDM and thus used a linear model to assess the association between HbA_1c_ and GDM^14^. The models were adjusted for maternal age (continuous), prepregnancy BMI (normal weight, overweight, obese), family history of diabetes (yes/no), and gestational week of blood collection (continuous). While maternal age and gestational week of blood collection were considered in the case-control matching, the matching was not exact and thus we adjusted for these factors to further remove any residual confounding. We tested for an interaction between HbA1c and pre-pregnancy BMI status (normal weight, overweight, obese). Women with GDM diagnosed before the first trimester HbA_1c_ measurement were excluded from the above analyses (n = 1).

Sensitivity analyses were performed excluding women who had GDM in a prior pregnancy (n = 5), women who had a hematologic disorder (n = 1), or obese women who smoked prior to pregnancy (n = 4).

Lastly, we examined for an association between the change in HbA_1c_ from enrollment at 8–13 weeks to visit 1 visit 2. These analyses excluded women with GDM diagnosed before enrollment (n = 1), visit 1 (n = 3) or visit 2 (n = 25), as appropriate.

#### First Trimester HbA_1c_ and GDM Prediction

We used a receiver-operating-characteristic (ROC) curves to evaluate the predictive ability of HbA_1c_ for GDM diagnosis. We used leave-one-out cross-validation to avoid overfitting the data with logistic regression models for ROC curves^15^. All models also accounted for the matched design in the case-control study by adjusting for the matching factors^16^. The following analyses excluded women with GDM diagnosed before the first trimester HbA_1c_ measurement (n = 1).

First, we estimated the sensitivity and specificity of first trimester HbA_1c_ by each 0.1% HbA_1c_ increase from 3.5% to 6.0% (15 to 42 mmol/mol). Confidence intervals around the sensitivity and specificity at each cutpoint were estimated using bootstrapping with 5000 repetitions, repeating the cross-validation for each repetition^17^. For each resampling repetition we identified the optimal HbA_1c_ cutpoint which maximized accuracy of classification based on sensitivity and specific using the Youden index (sensitivity + [specificity-1])^18^. The overall suggested optimal point corresponded to the mode of the distribution of identified cutpoints across the 5000 replicates.

Second, we estimated the value of using HbA_1c_ for prediction of GDM above and beyond conventional high-risk factors (i.e., maternal age, race-ethnicity, pre-pregnancy overweight or obesity, family history of diabetes, GDM in a prior pregnancy, and nulliparity).

All analyses were conducted using SAS version 9.4 (SAS Institute, Cary, NC) and p-values < 0.05 were considered significant.

## Results

The participant characteristics of the women with and without GDM are shown in Table 1. Women with GDM were more likely to be obese and have a family history of diabetes; as expected due to matching no differences between cases and controls were observed in race/ethnicity or age. At enrollment, 15.0% of GDM cases (n = 15) and 2.4% of non-GDM controls (n = 5) had an HbA_1c_ level ≥5.7%, corresponding to the cutpoint for prediabetes outside of pregnancy; this difference was not significantly different (P = 0.54).

**Table 1:** Baseline characteristics among women who developed gestational diabetes (GDM) and non-GDM controls, NICHD Fetal Growth Studies- Singletons (2009–2013).

Characteristic	Non-GDM Controls (n = 211)		GDM Cases (n = 100)		P
	n	(%)	n	(%)	
Age, y					0.63
<25	36	(17.1)	17	(17.0)	
25–34	121	(57.4)	56	(56.0)	
≥35	54	(25.6)	27	(27.0)	
Race-ethnicity					0.16
Non-Hispanic White	50	(23.7)	24	(24.0)	
Non-Hispanic Black	30	(14.2)	11	(11.0)	
Hispanic	80	(37.9)	40	(40.0)	
Asian/Pacific Islander	51	(24.2)	25	(25.0)	
Pre-pregnancy body mass index, kg/m^2^					<0.001
18.5–24.9	122	(57.8)	36	(36.0)	
24.9–29.9	56	(26.5)	30	(30.0)	
≥30.0	33	(15.6)	34	(34.0)	
Education					0.16
Less than high school	26	(12.3)	16	(16.0)	
High school or equivalent	23	(10.9)	15	(15.0)	
More than high school	162	(76.8)	69	(69.0)	
Marital status					0.15
Not married	45	(21.3)	14	(14.0)	
Married or living with partner	166	(78.7)	86	(86.0)	
Family history of diabetes					0.009
Yes	48	(22.8)	38	(38.0)	
No	163	(77.3)	62	(62.0)	
Parity and prior GDM					0.95
Nulliparous	95	(45.0)	45	(45.0)	
Parous, no prior GDM	113	(53.6)	53	(53.0)	
Parous, prior GDM	3	(1.4)	2	(2.0)	
Smoked prior to pregnancy					0.16
Yes	1	(0.5)	3	(3.0)	
No	210	(99.5)	97	(97.0)	
Hematologic disorders					0.32
Yes	0	(0.0)	1	(1.0)	
No	211	(100.0)	99	(99.0)	

Abbreviations: GDM, gestational diabetes.

### HbA_1c_ Profile Across Pregnancy

**Figure 1:**
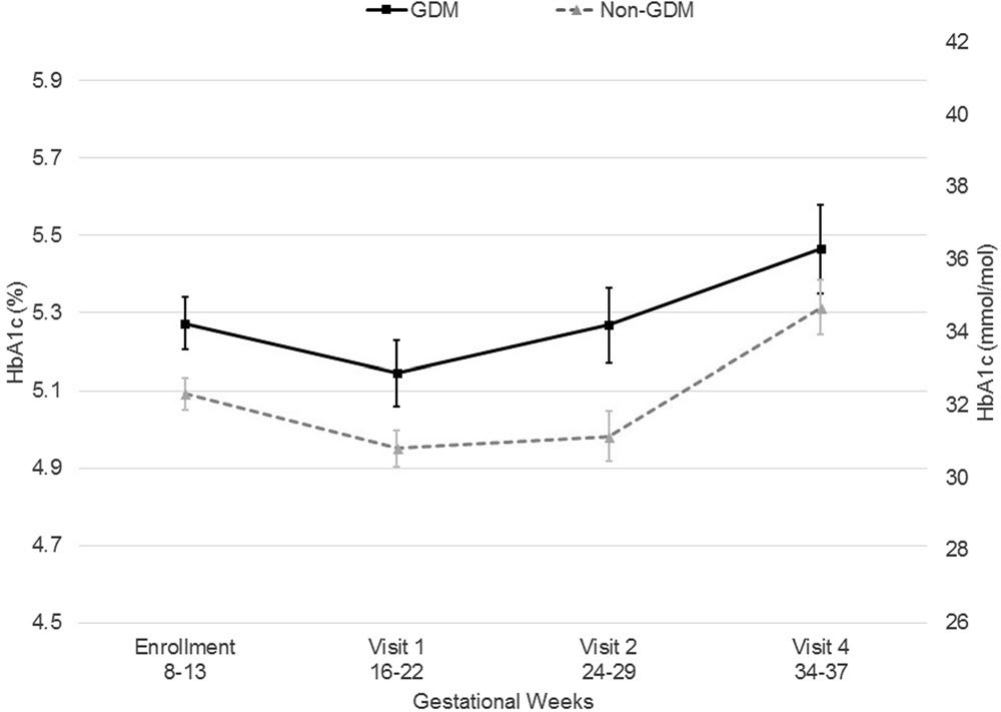
Longitudinal changes in HbA_1c_ across gestation among women with gestational diabetes (GDM) during pregnancy and their matched non-GDM controls, NICHD Fetal Growth Studies-Singletons (2009–2013). ^a^The difference between GDM and non-GDM was significant (P ≤ 0.03) at all gestational weeks. ^b^Data represented as mean ± 95% confidence limit. ^c^Data are presented by study visit. At enrollment (8–13 gestational weeks), 99.7% of the blood draws were within the targeted range. At visit 1 (16–22 weeks), 91.0% of the blood draws were within the targeted range. At visit 2 (24–29 weeks), 90.3% of the blood draws were within the targeted range. No blood was collected at visit 3. At visit 4 (34–37 weeks), 89.2% of the blood draws were within the targeted range.

The longitudinal changes in HbA_1c_ across gestation according to GDM status are shown in Fig. 1. HbA1c was significantly higher in GDM cases than controls throughout pregnancy (P < 0.03). Regardless of GDM status, HbA_1c_ tended to decrease into the second trimester, and then increase around the third trimester.

### Association between HbA_1c_ and GDM Risk

There was a significant linear association between HbA_1c_ at enrollment (8–13 weeks) and GDM risk (P = 0.001). HbA_1c_ was associated with a significant increased risk of GDM such that compared to women with median HbA_1c_ levels (5.2%; 33 mmol/mol), women with a first trimester HbA_1c_ of 5.7% (39 mmol/mol) had a GDM risk of 2.73 (95% CI 1.59, 4.66) times higher **(**Table 2**)**. The change in HbA_1c_ levels between enrollment and visit 1 was not associated with GDM risk independent of HbA_1c_ levels at enrollment. However, the change in HbA_1c_ between enrollment and visit 2 was significantly and positively associated with GDM risk independent of HbA_1c_ at enrollment (P = 0.04). Sensitivity analyses excluding obese women who smoked, had prior GDM, or a hematologic disorder yielded similar results [per 0.1% increase HbA_1c_: OR = 1.23 (95% CI 1.10, 1.38)].

**Table 2:** Odds of gestational diabetes (GDM) according to HbA_1c_ level at 8–13 weeks and the change in HbA_1c_ from 8–13 weeks and 16–22 and 24–29 weeks, NICHD Fetal Growth Studies- Singletons (2009–2013).

	Crude Model^b^		Adjusted Model^b,c^	
	OR (95% CI)	*P*	OR (95% CI)	*P*
HbA_1c_ at Visit 0^a^				
*Per 0*.*1%*	1.27 (1.14, 1.40)	<0.001	1.22 (1.09, 1.36)	<0.001
Change in HbA_1c_ from visit 0 to 1^a^				
*Per 0*.*1%*	1.10 (0.95, 1.16)	0.19	1.04 (0.90, 1.21)	0.57
Change in HbA_1c_ from visit 0 to 2^a^				
*Per 0*.*1%*	1.21 (1.01, 1.44)	0.03	1.21 (1.01, 1.45)	0.04

Abbreviations: CI, confidence interval.

^a^Visit 0: 8–13 weeks. Visit 1: 16–22 weeks. Visit 2: 24–29 weeks.

^b^Non-GDM controls matched to each GDM case on maternal age (±2 years), race/ethnicity (non-Hispanic White, non-Hispanic Black, Hispanic, Asian/Pacific Islander), and gestational week of blood collection (±2 weeks). Models additional adjusted for maternal age and gestational age at delivery to remove any remaining residual confounding with these factors.

^c^Model additionally adjusted for family history of diabetes and pre-pregnancy overweight and obesity.

### First Trimester HbA_1c_ and GDM Prediction

The sensitivity and specificity of HbA_1c_ at enrollment (8–13 weeks) for GDM is presented in Table 3. Sensitivity ranged from 96% (95% CI 91%, 100%) at an HbA_1c_ level of 3.5% (15 mmol/mol) to 12% (95% CI 1%, 27%) at an HbA_1c_ of 6.0% (42 mmol/mol). Specificity ranged from 10% (95% CI 1%, 23%) at an HbA_1c_ of 3.5% (15 mmol/mol) to 98% (95% CI 95%, 100%) at an HbA_1c_ of 6.0% (42 mmol/mol). There was suggestion that the optimal HbA_1c_ cutpoint was at 5.1% (32 mmol/mol) where the sensitivity was 47% (95% CI 34%, 60%) and the specificity was 79% (95% CI 62%, 88%). At an HbA_1c_ of 5.7% (39 mmol/mol), corresponding to the cutoff for prediabetes outside of pregnancy, the sensitivity was 21% (95% CI 8%, 36%) and the specificity was 95% (95% CI 91%, 99%).

**Table 3:** Sensitivity and specificity of HbA_1c_ at 8–13 weeks gestation and gestational diabetes diagnosis, NICHD Fetal Growth Studies-Singletons (2009–2013).

HbA_1c_		Sensitivity (95% CI)	Specificity (95% CI)
%	mmol/mol		
3.5	15	0.96 (0.91, 1.00)	0.10 (0.01, 0.23)
3.6	16	0.95 (0.90, 0.99)	0.11 (0.01, 0.26)
3.7	17	0.94 (0.88, 0.99)	0.13 (0.02, 0.29)
3.8	18	0.93 (0.87, 0.98)	0.15 (0.02, 0.33)
3.9	19	0.92 (0.84, 0.98)	0.18 (0.03, 0.36)
4.0	20	0.90 (0.82, 0.97)	0.21 (0.04, 0.40)
4.1	21	0.88 (0.79, 0.96)	0.25 (0.05, 0.45)
4.2	22	0.86 (0.75, 0.95)	0.29 (0.07, 0.50)
4.3	23	0.83 (0.71, 0.93)	0.33 (0.09, 0.54)
4.4	25	0.79 (0.66, 0.91)	0.39 (0.12, 0.59)
4.5	26	0.75 (0.62, 0.89)	0.44 (0.17, 0.64)
4.6	27	0.71 (0.57, 0.86)	0.50 (0.22, 0.69)
4.7	28	0.66 (0.53, 0.82)	0.56 (0.28, 0.74)
4.8	29	0.61 (0.48, 0.77)	0.63 (0.35, 0.78)
4.9	30	0.56 (0.43, 0.71)	0.69 (0.44, 0.82)
5.0	31	0.51 (0.39, 0.66)	0.74 (0.54, 0.85)
5.1	32	0.47 (0.34, 0.60)	0.79 (0.62, 0.88)
5.2	33	0.42 (0.29, 0.55)	0.83 (0.70, 0.91)
5.3	34	0.38 (0.24, 0.51)	0.87 (0.76, 0.93)
5.4	36	0.33 (0.20, 0.47)	0.90 (0.82, 0.95)
5.5	37	0.29 (0.15, 0.44)	0.92 (0.86, 0.96)
5.6	38	0.25 (0.11, 0.40)	0.94 (0.89, 0.98)
5.7	39	0.21 (0.08, 0.36)	0.95 (0.91, 0.99)
5.8	40	0.18 (0.05, 0.33)	0.97 (0.93, 0.99)
5.9	41	0.15 (0.03, 0.30)	0.97 (0.94, 1.00)
6.0	42	0.12 (0.01, 0.27)	0.98 (0.95, 1.00)

Abbreviations: CI, confidence interval.

We observed significant improvement (P = 0.04) to the base prediction model based on conventional risk factors (age, race/ethnicity, pre-pregnancy overweight and obesity, family history of diabetes, GDM in a prior pregnancy, and nulliparity; AUC = 0.59) with the inclusion of HbA_1c_ measured at enrollment (AUC = 0.65) (Supplementary Fig. S1).

## Discussion

In this prospective study among women without pre-existing medical conditions, we systematically examined HbA_1c_ measured across pregnancy starting in the first trimester and its relation with GDM risk. The risk of GDM increased in a profound linear fashion such that women with an HbA_1c_ of 5.7% (39 mmol/mol) compared to 5.2% (33 mmol/mol) had an almost three times higher risk of developing GDM. Furthermore, we observed a significant improvement in GDM prediction with the inclusion of first trimester HbA_1c_ over conventional risk factors alone. Thus, even in this cohort of low-risk women without pre-existing medical conditions, HbA1c measured in the first trimester improved GDM prediction.

We examined the longitudinal trends in HbA_1c_ levels across pregnancy among women who did and did not develop GDM. Women without GDM had lower first trimester HbA_1c_ levels than women who went on to develop GDM; however, both groups followed similar patterns across pregnancy; HbA_1c_ decreased slightly from the first to the second trimester and then tended to increase in the third trimester. This is intuitive and in line with the high erythrocyte turnover in pregnancy^19^, and the decrease in insulin sensitivity with increasing gestation^20^. While our findings are similar to other studies, such as Nielsen et. al who reported that levels decreased between two measurements from early to later in pregnancy^21^, we provide longitudinal data across pregnancy to show the complete pattern in each trimester. Another study observed similar patterns across pregnancy, but each time point was based only on cross sectional measurements with one observation per participant^22^.

Outside of pregnancy, HbA_1c_ has been shown to be a useful biomarker for diagnosing type 2 diabetes and monitoring glucose control among individuals with diabetes. Its current application in pregnancy has been limited to screening for overt type 2 diabetes and it remains unclear if it has utility for GDM screening. We observed that HbA_1c_ provided a clinically meaningful improvement in GDM prediction over conventional high-risk factors with a 0.06-point improvement in the AUC. At the suggested ‘optimal’ cutpoint of 5.1% (32 mmol/mol), the sensitivity remained relatively low at 47% and the specificity was moderately high at 79%. This optimal cutpoint maximizes the effectiveness of the test, but may not be ideal for the sole purpose of GDM diagnosis given that we have existing diagnostics based on the OGTT and the purpose of measuring HbA_1c_ would only be an earlier diagnosis. Nonetheless, at a cutpoint of 5.7% (39 mmol/mol), the current cutpoint for prediabetes outside of pregnancy, the sensitivity was 21%, but the specificity was very high at 95%, which is very similar to estimates reported in a prior study of 13% and 94%, respectively^6^. There are two important things to note from our findings. First, the low sensitivity at higher HbA_1c_ levels suggests that HbA_1c_ may not be a good substitute for a second trimester OGTT, which tests the acute response to the glucose challenge and how women respond to the increased insulin resistant environment of late pregnancy. However, more importantly, the high specificity at 5.7% (39 mmol/mol) suggests that with this threshold few low-risk women, who otherwise would not receive early screening, would be incorrectly diagnosed by an elevated *first trimester* HbA_1c_ level. This presents a unique opportunity for earlier interventions in these women which would be ideal as GDM is associated with adverse pregnancy outcomes such as macrosomia^23^. While it is plausible that with an earlier intervention these risks might be minimized, future studies evaluating early intervention based on elevated first trimester HbA_1c_ are essential to determine its utility.

Another important finding of our study with biological relevance was that with increasing levels of HbA_1c_, GDM risk increased significantly and in a linear fashion. Because HbA_1c_ reflects glucose levels in the prior two to three months, we interpret these results to suggest that hyperglycemia even within women without pre-pregnancy diabetes may be relevant for the development of GDM. This finding is in line with prior studies on preconception diet which have observed substantial increased risks for GDM with poor dietary quality, further supporting the hypothesis that preconception improvements in glucose function may aid in GDM prevention^24^. It is important to note, however, that as we make inferences from our findings to the preconception period, the findings from first trimester HbA_1c_ could also reflect small changes in the first few weeks of pregnancy, therefore confirmation of preconception aberrations in HbA_1c_ levels among women who develop GDM need to be verified in future studies.

One strength of our study was that HbA_1c_ levels were measured among all women regardless of their clinical status. Some prior studies on HbA_1c_ and GDM have been limited only to women who have an elevated risk for GDM at the start of their pregnancy. In accordance with current American Diabetes Association recommendations^25^, we considered women to have had overt diabetes if their first trimester HbA_1c_ was ≥6.5% (48 mmol/mol) and excluded them from our analyses. GDM screening and diagnosis was completed according to standard clinical practice and the Carpenter and Coustan criteria were used to classify women with GDM after review of their medical records^13^. In addition, our study sample represented multiple race-ethnic groups from across the U.S., furthering the generalizability of our findings, although our findings may not be fully generalizable to women outside of the U.S. Also, HbA_1c_ was measured according to the National Glycohemoglobin Standardization Program (NGSP) approved diagnostic test. We excluded participants identified as having hemoglobin variants (n = 6), for whom the TOSOH G7 does not perform well and HbA_1c_ values can be underestimated^26^.

One limitation of our study was that the sample size was somewhat small at 321 participants (107 women with GDM and 214 matched controls), however, this was based on the larger underlying cohort of women from across the United States providing adequate power to address the research question. Nonetheless, the sample size did preclude us from creating training and confirmation datasets for our predication models. However, we applied leave-one-out cross-validation to avoid overfitting the model. Nonetheless, inferences regarding GDM diagnostics based on the ROC curves can only be treated as preliminary and require replication. In addition, the controls were matched to the GDM cases according to their age and race-ethnicity. While the matching was appropriately accounted for in our methods and the 6-point AUC difference between the models with and without HbA_1c_ is valid, the overall AUC statistics are likely underestimated as the matching factors of age, race/ethnicity, which are strong risk factors for GDM, are not contributing to the AUC. Also, matching on all potential confounders is not possible, and therefore the cases and controls were imbalanced in regard to pre-pregnancy BMI and family history of diabetes, but this was accounted for through adjustment. Additionally, not all women had their blood drawn during the targeted window for each visit; however, for the enrollment visit, where the majority of our conclusions are based, 99.7% of data were within the targeted range and are considered first trimester measurements. Lastly, the NICHD Fetal Growth Studies inclusion criteria were restricted to women without major chronic diseases and thus these results may not be generalizable to a high-risk population; however, early screening for overt type 2 diabetes is already completed in high risk women our findings suggest that even in low-risk women early dysfunction in glucose metabolism may be observed.

In conclusion, our comprehensive findings suggest potential important clinical utility of HbA_1c_ measurement in the first trimester of pregnancy, even among low-risk women. While our findings require replication, GDM prediction was significantly improved with the inclusion of HbA_1c_ over conventional risk factors suggesting that it could be used to improve early risk-stratification and screening in women with elevated levels. Furthermore, our findings suggest that hyperglycemia even among women without pre-pregnancy diabetes may be important for the development of GDM. These findings can be utilized to inform future intervention studies.

## Electronic supplementary material

Supplementary Figure S1
