# Supplementary material for: HbA1c Measured in the First Trimester of Pregnancy and the Association with Gestational Diabetes
Source: Sci Rep. 2018 Aug 16;8:12249. doi: 10.1038/s41598-018-30833-8 (PMC6095876; doi:10.1038/s41598-018-30833-8)

HbA1c Measured in the First Trimester of Pregnancy and the Association with Gestational Diabetes

Stefanie N. Hinkle, Michael Y. Tsai, Shristi Rawal, Paul S. Albert, Cuilin Zhang

Supplementary Material

HbA1c Measured in the First Trimester of Pregnancy and the Association with Gestational Diabetes

Stefanie N. Hinkle, Michael Y. Tsai, Shristi Rawal, Paul S. Albert, Cuilin Zhang

Supplementary Figure S1. Receiver operating characteristic (ROC) curves and area under the curve (AUC) statistics for the prediction of gestational diabetes (GDM) using conventional risk factors only (age, race/ethnicity, family history of diabetes, nulliparity, prior GDM, and pre-pregnancy overweight and obesity) or conventional risk factors and the addition of first trimester HbA_{1c}.

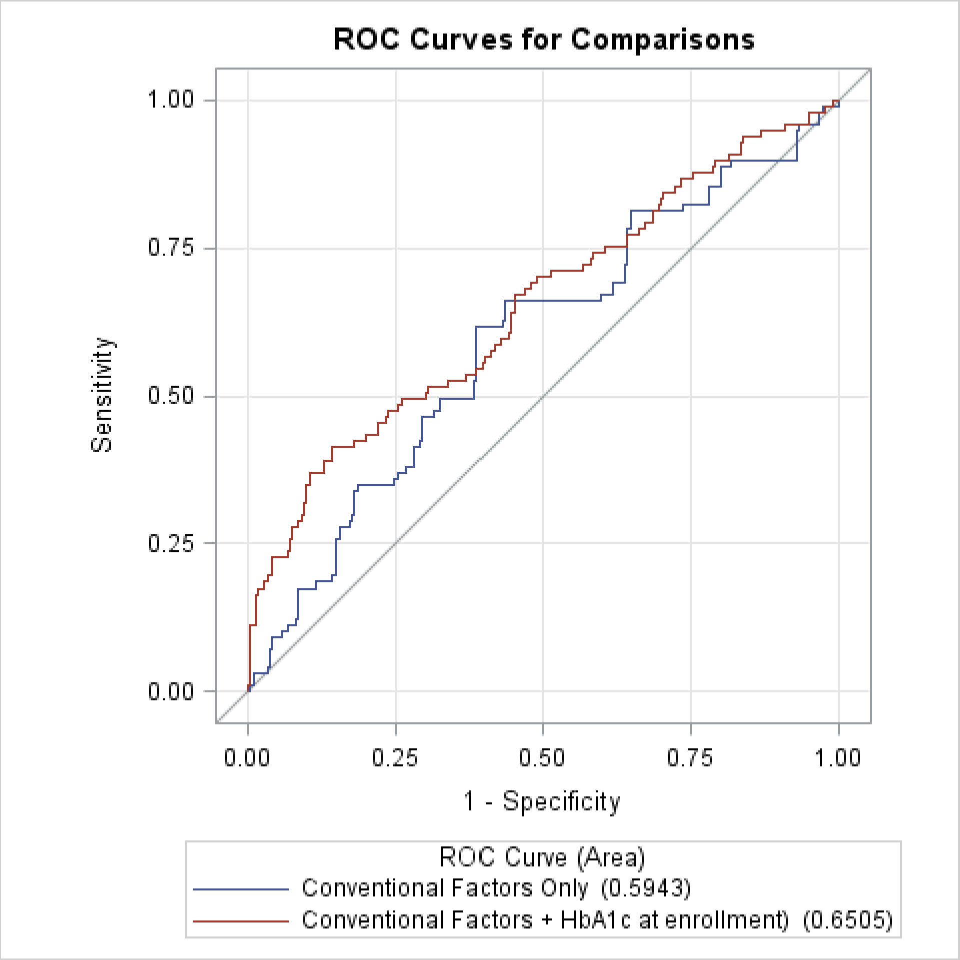

Supplement: Supplementary file 1 — Supplementary Figure S1 [file 41598_2018_30833_MOESM1_ESM.pdf]
